# ACmix-Swin Deep Learning of 4-Day-Old *Apis mellifera* Larval Transcriptomes Reveals Early Caste-Biased Regulatory Hubs

**DOI:** 10.3390/genes17010017

**Published:** 2025-12-25

**Authors:** Peixun Gong, Jinyou Li, Weixue Tian, Xiang Ding, Runlang Su, Dan Yue

**Affiliations:** 1 College of Animal Science and Technology, Yunnan Agricultural University, Kunming 650201, China; 2 Faculty of Computing & Data Sciences, Boston University, Boston, MA 02215, USA; 3 School of Mechanical and Electrical Information, Yiwu Industrial and Commercial College, Jinhua 322000, China; 4 University of Chinese Academy of Sciences, Beijing 100049, China

**Keywords:** *Apis mellifera*, RNA-seq, caste differentiation, deep learning, ACmix-Swin

## Abstract

Background/Objectives: Early larval development is critical for caste and sex differentiation in honeybees. This study investigates molecular divergence in 4-day-old *Apis mellifera* larvae and introduces a customized deep learning model for hub-gene discovery. Methods: Genome-guided RNA-seq, DEGs, WGCNA, and splicing analyses were integrated. A hybrid convolution–attention model, ACmix-Swin, combined with WGAN-GP augmentation, was developed to classify larvae and prioritize caste-biased genes. Selected genes were validated by qPCR. Results: Significant caste- and sex-specific divergence was detected in cuticle formation, hormone metabolism, and reproductive signaling. ACmix-Swin achieved the highest accuracy among baseline models and consistently identified key regulators, including *Vg*, *LOC725841*, *LOC412768*, and *LOC100576841*. qPCR confirmed RNA-seq trends. Conclusions: Caste- and sex-specific transcriptional programs are established early in larval development. The ACmix-Swin framework provides an effective strategy for high-dimensional transcriptome interpretation and robust hub-gene identification.

## 1. Introduction

Bees are essential pollinators, and their colonies consist of queens, drones, and workers, each performing distinct biological roles [1,2,3,4,5,6]. These castes differ in morphology as well as in epigenetic and molecular regulatory features [1,3]. The queen is the only reproductive female, drones function mainly in mating, and workers—sterile females—carry out brood care, foraging, nest construction, and colony defense [4,5,6]. Honeybee development proceeds through embryonic, larval, pupal, and adult stages. The larval period is a highly dynamic phase involving rapid growth and extensive transcriptomic and epigenetic remodeling that underlies caste differentiation. Before day three, larvae are fed royal jelly, allowing female larvae to retain the potential to develop into either workers or queens [7]. The fourth larval day is a key transition point at which metabolic reprogramming and major shifts in gene regulation occur. Many caste-related, immune, and metabolic pathways show their strongest changes between days 3 and 5, making day four an informative stage for studying developmental regulation [8,9,10,11]. Nevertheless, despite these technical constraints, these studies consistently demonstrated that 4-day-old larvae represent a critical—and effectively a “golden”—developmental window for investigating honeybee caste differentiation, during which caste-specific transcriptional programs are robustly established [12]. Honeybee caste differentiation has been extensively investigated at the transcriptomic level, revealing the complex molecular mechanisms underlying phenotypic plasticity. Previous RNA-seq studies have demonstrated that thousands of genes are differentially expressed between queen- and worker-destined larvae during critical developmental windows [13,14]. These studies indicate that caste-specific developmental trajectories are established through coordinated changes in gene expression involving hormonal signaling, metabolic reprogramming, and epigenetic regulation [12,15]. More recently, advances in sequencing technologies have uncovered additional layers of regulation, including alternative splicing, poly(A) tail dynamics, and RNA modifications such as m6A methylation, all of which contribute to the formation of caste-specific transcriptomic landscapes [15]. Honeybee caste differentiation is a classical model for understanding developmental plasticity and has been extensively investigated at the transcriptomic level. Early microarray-based studies first revealed differential gene expression between developing queens and workers, identifying metabolic enzymes and regulatory factors as early molecular markers of caste differentiation [16]. Subsequent RNA-seq analyses systematically compared queen, worker, and drone larvae, demonstrating that reproductive, metabolic, and developmental pathways begin to diverge already at the larval stage [12,17]. Subsequent work further characterized the transcriptomic signatures of social interactions underlying caste development, emphasizing the dynamic interplay between intrinsic gene regulatory programs and extrinsic nutritional signals [17]. In the context of honeybee caste differentiation, extensive studies have already identified numerous caste-associated genes and pathways. For example, Vitellogenin (*Vg*) has been repeatedly recognized as a central regulator linking reproduction, metabolism, and social behavior, and is consistently enriched in queens relative to workers and drones. However, despite this rich body of work, there is still no consensus on which genes constitute the core regulators that most directly distinguish queen, worker, and drone phenotypes [10]. Most existing studies emphasize differential expression or functional enrichment, whereas the relative contribution and discriminative importance of individual genes across castes remain insufficiently resolved. This unresolved question motivates the use of model-based feature prioritization approaches, which aim to rank genes according to their contribution to caste discrimination rather than expression change alone [12].

Advances in sequencing technologies have greatly increased transcriptomic data volume, yet computational methods have not kept pace [18]. Modern transcriptomes are multidimensional and heterogeneous, presenting challenges for data interpretation [19,20]. Although public databases contain abundant transcriptome resources, only a small fraction is deeply analyzed [20]. Conventional statistical approaches identify differentially expressed genes but often fail to pinpoint key regulators driving phenotypic divergence [21,22,23]. Machine learning (ML) offers improved performance in handling large, complex datasets and integrating heterogeneous information [24,25], and automated ML further reduces technical barriers [26].

Deep learning (DL) has shown superior predictive accuracy compared with traditional ML in genomic applications [27,28,29], but small sample sizes often lead to overfitting. Generative adversarial networks (GANs) address this limitation by producing synthetic synthetic data improving model stability and performance [30,31]. GANs generate latent-space data through adversarial optimization and have been widely adopted in bioinformatics [31].

In this study, the WGAN-GP algorithm was applied to generate synthetic transcriptomic profiles for 4-day-old larvae of the three honeybee castes [32]. A hybrid architecture combining Swin-Transformer and CNN models was then constructed by integrating both original and GAN-generated datasets [33,34]. This fusion-based framework enabled efficient feature extraction and accurate classification of caste-specific transcriptomic patterns.

## 2. Materials and Methods

### 2.1. Sample Collection

Transcriptome datasets of 4-day-old Italian honeybee (*Apis mellifera ligustica*) larvae were retrieved from the NCBI Sequence Read Archive (SRA). These datasets were originally generated and published in previous transcriptomic studies investigating caste differentiation in honeybee larvae. In the present study, we reanalyzed these publicly available data using an updated reference genome and advanced analytical frameworks. For each caste at each developmental stage, six biological replicates were available, including 4-day-old workers (SRS1263211), queens (SRS1263243), and drones (SRS1263256) [12]. For the qPCR experiment, larvae were collected from A. mellifera colonies maintained near Yiwu Industrial & Commercial College. Colonies were kept at approximately 34 °C and 70% relative humidity without additional environmental manipulation. After collection, larval tissues were homogenized, filtered, and stored at −20 °C.

Total RNA was extracted using the Flash Pure Total RNA Micro Kit (catalog no. R516-PRL, Beijing, China) from embryonic samples preserved in PRL lysis buffer, following the manufacturer’s instructions. Subsequent qPCR assays were performed by Anhui Gaohes Biological Co., Ltd., AnQing, China.

### 2.2. Alignment and Quality Control of RNA-Seq Data

Quality control of the cleaned sequencing data was performed using FastQC. Raw reads were processed with Fastp (v0.23.2) for adapter removal and quality trimming, using the following parameters: automatic detection of paired-end adapters, Q20 trimming, filtering of reads with >40% low-quality bases or >5% ambiguous bases, and retention of reads ≥50 bp in length. Clean reads were aligned to the *Apis mellifera* reference genome (Amel_HAv3.1) using Hisat2 (v2.2.1) [35]. Hisat2 was run with default settings, including a 20 bp seed length, allowance for soft clipping, mismatch penalties of 6 and 2, and reporting up to five valid alignments per read.

SAM files were converted to sorted BAM files using Samtools (v1.6) [36]. Gene-level read counts were then generated with FeatureCounts [37]. Differential expression analysis was conducted using the DESeq2 algorithm integrated within Trinity (v2.15.1) [38,39]. *p*-values were adjusted using the Benjamini–Hochberg false discovery rate (FDR) method, and genes with FDR < 0.05 and |log_2_FC| > 1 were considered significantly differentially expressed.

### 2.3. GO Enrichment Analysis of Differentially Expressed Genes and Alternatively Spliced Genes

GO annotation was performed using the longest CDS-derived protein sequences from the reference genome. Functional annotation was conducted with emapper.py (v2.1.10) [40] based on the EggNOG database [41]. The resulting annotation file was then converted into an org.db format, and GO enrichment analysis was carried out using the clusterProfiler package (v4.4) in R.

### 2.4. Alternative Splicing Analysis

Alternative splicing analysis was performed using SUPPA (v2.4) together with the reference genome annotation file (GTF) [42,43]. SUPPA was used to quantify five types of alternative splicing events (SE, MXE, A5, A3, RI), and transcript abundance was estimated with Salmon (v1.10.1). For each phenotype group (workers, drones, and queens), six biological replicates were used to calculate PSI values for all splicing events, and ΔPSI was computed to assess differences between groups. Significant differential splicing events were defined as those with ∆PSI ≥ 0.1 and *p* < 0.05 after FDR correction.

### 2.5. WGCNA Analysis

After removing outlier samples based on pairwise correlation analysis and hierarchical clustering, a weighted gene co-expression network was constructed using the WGCNA R package (v1.73) (https://cran.r-project.org/web/packages/WGCNA/index.html, accessed on 4 December 2025). The network was built with the following parameters: networkType = “signed”, mergeCutHeight = 0.25, minModuleSize = 30, and deepSplit = 2. Automatic block-wise module detection was applied, and a data-driven soft-thresholding power was selected to approximate scale-free topology. Module eigengenes (MEs) were calculated and correlated with experimental conditions to identify phenotype-associated modules. Gene–gene regulatory networks were then extracted from the selected modules.

### 2.6. Model Construction

To address the limitations imposed by high-dimensional transcriptomic data with small sample sizes, an integrated data augmentation strategy based on the Wasserstein GAN with Gradient Penalty (WGAN-GP) framework was employed. The WGAN-GP generator consisted of two fully connected layers (hidden dimension = 128; output dimension matching the number of transcriptomic features), whereas the discriminator was implemented using a three-layer architecture with spectral normalization to ensure training stability. Training parameters included a noise vector dimension of 64, a gradient penalty coefficient (λ_gp) of 10, a critic–generator update ratio of n_critic = 5, and the Adam optimizer (learning rate = 1 × 10^−4^, β_1_ = 0.0, β_2_ = 0.9).

Following adversarial training, multiple augmentation strategies were integrated to increase sample diversity and maintain biological plausibility. The augmented dataset consisted of WGAN-GP synthetic samples (30% of total augmentation), SMOTE-based interpolation (15%), Gaussian-noise perturbation (35%, with noise scaled to 0.05–0.15 of the feature-wise standard deviation), and Mixup combinations (20%, using a Beta(0.4, 0.4) mixing coefficient). To prevent synthetic samples from deviating excessively from the true data distribution, only WGAN-GP outputs with distances less than twice the median within-class distance were retained.

**Figure 1:**
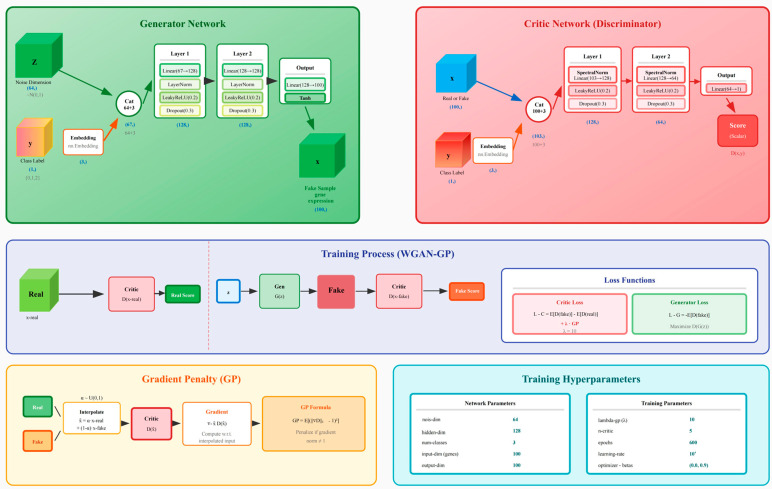
Illustrates the complete WGAN-GP architecture, including the generator network (green), critic network (red), training process (blue), gradient penalty computation (yellow), and training hyperparameters (cyan).

After filtering and integration, each phenotype category was expanded to 50–100 samples to meet the requirements of downstream deep learning model training. A schematic overview of the workflow is presented in Figure 1.

In model construction, a hybrid deep learning framework named ACmix-Swin was designed, integrating the strengths of CNN and Swin-Transformer architectures to capture both local gene co-expression patterns and global regulatory signals [44,45,46]. Input features were first processed through two fully connected embedding layers with LayerNorm, GELU activation, and a dropout rate of 0.15 to generate unified feature representations.

The core ACmix-Swin module consists of parallel convolutional and self-attention branches. The self-attention branch adopts a Swin-style windowed multi-head mechanism, which incorporates relative positional bias to model fine-grained regional dependencies. The attention computation is defined as:

(1) $$Attention\left(Q,K,V\right)=softmax\left(\frac{QK^{T}}{\sqrt{d_{k}}}+B\right)V$$

The convolutional branch employed depthwise separable convolutions (kernel size = 3, groups = embed_dim) followed by pointwise convolutions to extract local expression features, together with BatchNorm and GELU activation. Outputs from the convolutional and self-attention branches were dynamically fused through a set of learnable parameters. The fused representation was subsequently processed by LayerNorm and adaptive average pooling before entering the classification head (dropout = 0.3). The final prediction of the three phenotypic classes (workers, drones, and queens) was obtained by modulating the contributions of the two branches using learnable weights α and β.

(2) $$Output=\alpha \cdot {Attention}_{branch}+\beta \cdot {Conv}_{branch}$$

**Figure 2:**
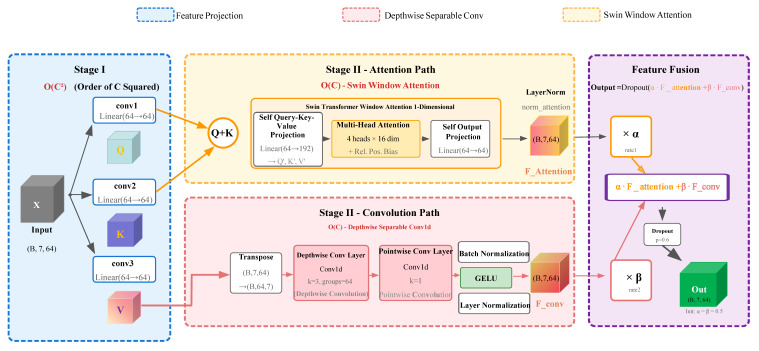
Illustrates the detailed implementation of the ACmix-Swin1D hybrid architecture. The architecture comprises three main stages: Stage I performs feature projection, transforming the input (B, 7, 64) into Q, K, V projections; Stage II consists of two parallel branches—the attention path employs Swin window attention for global dependency modeling, while the convolution path uses depthwise separable convolution for local feature extraction; finally, features are fused through learnable weights α and β, outputting dimensions of (B, 7, 64). (Q+K for attention computation and V for the convolution path.).

The hybrid architecture of the ACmix-Swin model is illustrated in Figure 2.

Model training was performed using the AdamW optimizer (learning rate = 1 × 10^−4^; weight decay = 1 × 10^−3^), together with cosine annealing scheduling and a warmup phase during the first 10 epochs. Cross-entropy loss with label smoothing (smoothing = 0.05) was applied, and Mixup augmentation (α = 0.2) and gradient clipping (max_norm = 1.0) were incorporated to reduce overfitting. Training was run for a maximum of 300 epochs, and an early-stopping criterion based on validation loss (patience = 50) was used to ensure stable convergence. Core model hyperparameters included an embedding dimension of 64, eight attention heads, a window size of 7, and a dropout rate of 0.6.

To identify key regulatory genes, feature attribution was computed using the Gradient × Input method, in which gradients of the model output with respect to the input gene expression values are obtained by backpropagation and multiplied element-wise with the input to estimate gene importance, defined as:

(3) $${Importance}_{i}=\left|\frac{\partial Loss}{\partial x_{i}}\times x_{i}\right|$$

To identify class-specific marker genes, phenotype-specific importance scores were further computed. Gene importance derived from the deep learning model (DL score) was integrated with WGCNA topological measures—gene significance (GS) and module membership (MM)—to construct a combined scoring framework, defined as:

(4) $${Combined\backslash }_{score}=w_{DL}\times {DL\backslash }_{score}+\left(1-w_{DL}\right)\times {WGCNA\backslash }_{score}$$

In this framework, wDLwDL controls the relative contribution of deep learning–derived importance and network topological metrics (e.g., wDL = 1.0 wDL = 1.0 indicates reliance on DL scores only, whereas wDL = 0.5 wDL = 0.5 represents equal weighting of DL and WGCNA). In this study, wDL = 1.0 wDL = 1.0. Based on the combined scores, the top 20 overall hub genes and the top 10 phenotype-specific hub genes for each caste were selected.

Model performance was systematically evaluated using accuracy, precision, recall, F1-score, and confusion matrices. All model training and score calculations were implemented in the PyTorch(v2.0) framework and accelerated using NVIDIA GPUs. The model implementation used in this study has been made publicly available on GitHub at: https://github.com/surunlang-creator/4dlarve-DLmodel, (accessed on 6 December 2025).

## 3. Results

### 3.1. RNA-Seq Overview and Quality Assessment

RNA sequencing was performed on all samples using the Illumina platform, generating an average of 3.9 Gbp of raw data per sample. After stringent quality filtering, approximately 3.85 Gbp of high-quality clean reads were retained for downstream analyses. Quality assessment indicated that both Q20 and Q30 values reached 100%, demonstrating excellent sequencing accuracy, and the mean GC content was 38.00%, consistent with expectations for the honeybee genome (Supplemental Table S1). Clean reads were aligned to the *Apis mellifera* reference genome using HISAT2, with more than 88% of reads uniquely mapped to a single genomic location. Sliding-window density analysis further confirmed the consistency and reliability of the mapping results. Across all 18 samples, a total of 645 million clean reads were uniquely mapped, yielding an overall average mapping rate of 94.47% (Supplemental Table S2).

### 3.2. Principal Component Analysis (PCA) of Samples

**Figure 3:**
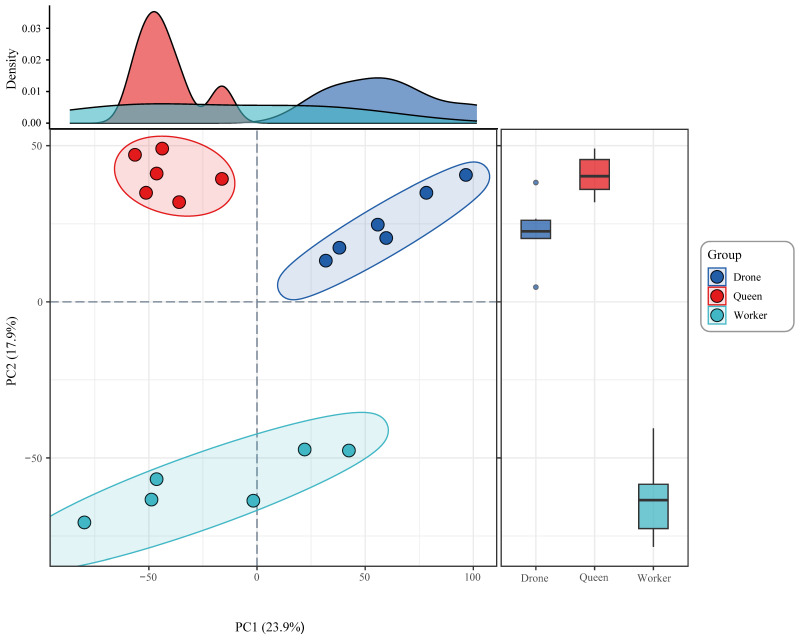
PCA and hierarchical clustering reveal clear caste-specific transcriptomic separation among drone, queen, and worker larvae.

Dimensionality reduction and clustering assessment of the expression matrix were performed using the scikit-learn library in Python(v3.10). Standardized gene expression values were first obtained through z-score normalization (sklearn.preprocessing.StandardScaler), followed by principal component analysis (PCA; sklearn.decomposition.PCA). The first two principal components (PC1 and PC2) accounted for the majority of variance among samples. The PCA projection revealed clear separation among queen, worker, and drone samples, while biological replicates within each group were tightly clustered. Density distributions along the axes illustrated overall variation patterns on PC1 and PC2, and accompanying boxplots further confirmed distinct expression characteristics across the three castes. Ellipses denote the 95% confidence intervals for each caste group (Figure 3).

### 3.3. Traditional Analysis of Differentially Expressed Genes (DEGs)

**Figure 4:**
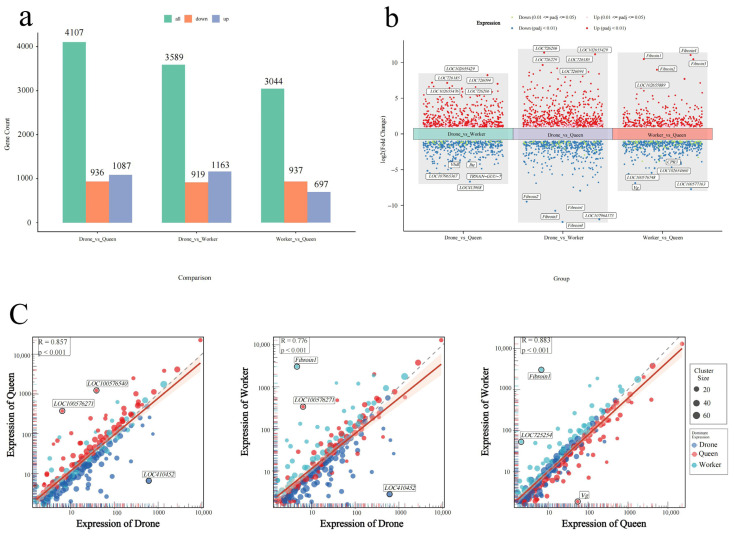
(**a**) Differentially expressed genes (DEGs) among drones, queens, and workers. (**b**) Volcano of differentially expressed genes among drone, worker, and queen larvae. (**c**) Gene Expression Correlation Analysis Among Bee Larval Castes via K-*means Clustering* (A–C) Pairwise comparison of cluster-level gene expression patterns between Drone, Queen, and Worker larvae at 4 days post-hatching. Each point represents a gene cluster (n = 200 clusters from 5000 high-variance genes). Correlation coefficients (R) and significance (*p*-value) are shown. Outlier genes (labeled) indicate caste-specific expression signatures. Diagonal dashed line (y = x) indicates equal expression.

In the comparison between drones and queens, 4107 differentially expressed genes (DEGs) were identified, including 1087 upregulated and 936 downregulated genes. A total of 3589 DEGs were detected between drones and workers (1163 upregulated and 919 downregulated), while 3044 DEGs were found between workers and queens (697 upregulated and 937 downregulated) (Figure 4a). Overall, drone expression profiles showed greater divergence from the two female castes, reflecting strong sex-related regulatory differences. In contrast, workers and queens exhibited fewer transcriptional differences, consistent with their shared female developmental trajectory.

Volcano plots illustrated the genome-wide expression patterns across castes (Figure 4b). In drone–queen comparisons, genes involved in cuticle formation (*LOC102655429*, *LOC726185*) and hormone regulation (*Jhe*) displayed pronounced differential expression. In drone–worker comparisons, fibroin-related genes (*Fibroin1–4*) were strongly downregulated in drones, indicating more active secretory functions in worker larvae. In worker–queen comparisons, reproductive and metabolic genes such as *CPR3* and *Vg* were markedly upregulated in queens.

KNN-based unsupervised clustering further supported these trends (Figure 4c). Female individuals (workers and queens) formed a tight cluster (R = 0.883), whereas drones were clearly separated from both female groups (R = 0.776–0.857). Key caste-associated genes included *Fibroin1* (higher in drones/queens) and *Vg* (lower in workers), suggesting that sex- and caste-related regulatory programs are already established at early larval stages.

### 3.4. Differential Gene Heatmap (DEGs)

**Figure 5:**
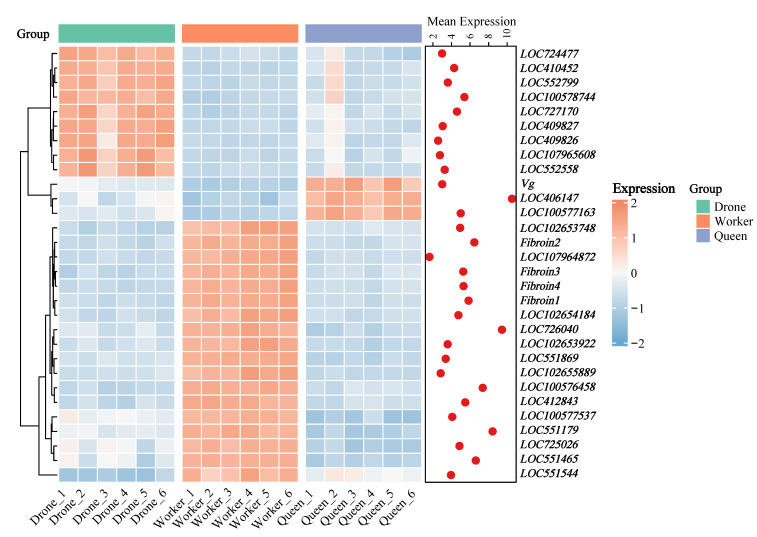
Expression patterns of the top 30 differentially expressed genes (DEGs) across drones, workers, and queens.

The expression patterns of the top 30 most significant DEGs across drones, workers, and queens were visualized using a heatmap (Figure 5). Hierarchical clustering revealed clearly separated transcriptomic clusters for the three castes. Workers showed consistently high expression for most DEGs, whereas drones and queens displayed lower or caste-specific patterns, indicating strong transcriptional differentiation.

Fibroin-related genes (*Fibroin1–4*) were strongly upregulated in workers, consistent with their roles in nest construction. In contrast, queens showed marked upregulation of *Vg* (Vitellogenin), which was nearly absent in workers and drones, highlighting its importance in queen reproduction and metabolic regulation.

Drone-specific high-expression genes included *LOC724477*, *LOC410452*, *LOC552799*, *LOC100578744*, *LOC727170*, *LOC409827*, *LOC409826*, *LOC107965608*, and *LOC552558*, all showing low expression in workers and queens. Worker-enriched genes included *LOC102653748*, *Fibroin2*, *LOC107964872*, *Fibroin3*, *Fibroin4*, *Fibroin1*, *LOC102654184*, *LOC726040*, *LOC102653922*, *LOC551869*, *LOC102655889*, *LOC100576458*, *LOC412843*, *LOC100577537*, *LOC551179*, *LOC725026*, *LOC551465*, and *LOC551544*. Queen-specific expression was observed mainly for *Vg*, *LOC406147*, and *LOC100577163* (Figure 5). These gene sets provide meaningful candidates for understanding the molecular mechanisms underlying honeybee caste differentiation.

### 3.5. GO and KEGG Analysis of Differentially Expressed Genes

**Figure 6:**
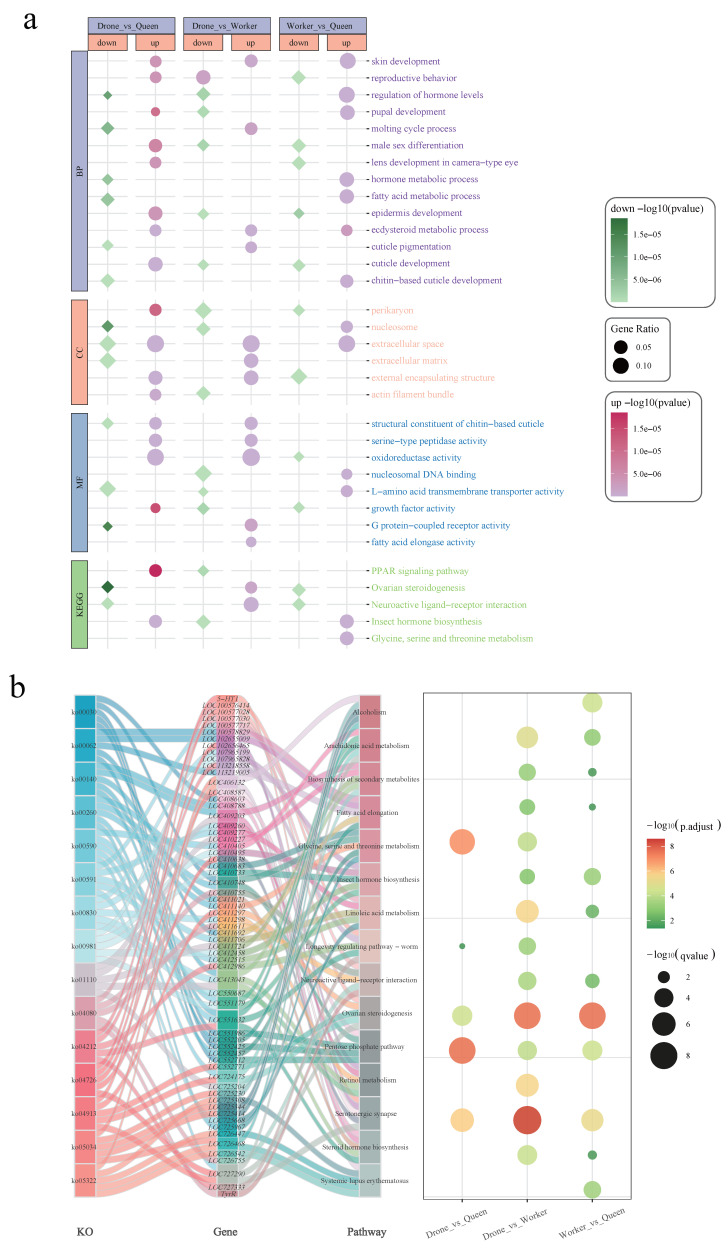
KEGG and GO Analysis. (**a**) GO and KEGG enrichment analysis of differentially expressed genes among three castes of honeybee. (**b**) KEGG pathway enrichment and gene-pathway relationships revealed by Sankey diagram and bubble plot of three castes of honeybee.

GO and KEGG enrichment analyses revealed clear functional divergence among the three castes (Figure 6a). Drones showed reduced expression in cuticle and reproductive pathways compared with queens but higher expression of mating-related genes, indicating a bias toward sex-specific behavioral regulation. Workers were enriched in pupal development, fatty acid metabolism, hormone metabolism, and lipid processing, suggesting early activation of developmental and metabolic programs linked to their labor roles. GO CC terms highlighted differences in exoskeleton and extracellular matrix organization, while MF terms were enriched for chitin-binding, oxidoreductase activity, and amino acid transport. KEGG analysis indicated that queens emphasized reproductive metabolism and neuroregulation, whereas drones were enriched in fundamental metabolic pathways. The KO–gene–pathway relationships shown in the Sankey diagram (Figure 6b) revealed major enrichment in amino acid metabolism, fatty acid elongation, and hormone biosynthesis, particularly in drone–queen and worker–queen comparisons. Some genes associated with reproductive signaling and neural pathways further reflected the transcriptional specialization of queens. Overall, queens were enriched in reproductive and hormonal pathways, workers in structural and metabolic processes, and drones in amino acid and lipid metabolism, forming the functional basis of caste differentiation.

### 3.6. Deep Learning Analysis

#### 3.6.1. WGAN-GP Analysis

**Figure 7:**
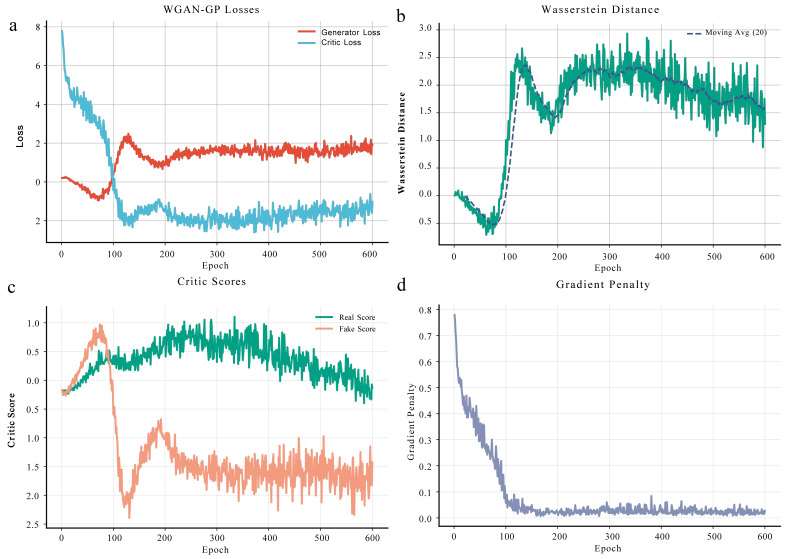
Training dynamics and convergence evaluation of WGAN-GP for transcriptomic data augmentation. (**a**) Training Loss Dynamics. (**b**) Wasserstein Distance Over Training. (**c**) Real vs. Generated Sample Scores. (**d**) Gradient Penalty Over Epochs.

To address the limitation of small sample size in transcriptomic analyses, the ACmix-Swin framework first applies Wasserstein GAN with Gradient Penalty (WGAN-GP) to augment all differentially expressed gene profiles, expanding each original sample into 100 synthetic samples to improve classification stability and generalization. The GAN was trained for 600 epochs, and four key indicators were used to assess training stability (Figure 7).

The generator and discriminator losses reached equilibrium after ~200 epochs, with generator loss stabilized at 1.5–2.0 and discriminator loss at −1.5 to −2.5 (Figure 7a), indicating stable adversarial dynamics without oscillation or mode collapse. The Wasserstein distance gradually decreased and stabilized at 1.5–2.0 (Figure 7b), and its 20-epoch moving average showed a consistent convergence trend, demonstrating that the generator effectively approximated the true data distribution. Discriminator scores also showed clear separation, with real samples centered around 0.4 and generated samples around –1.7 (Figure 7c), reflecting strong discriminative ability and suggesting room for further refinement of synthetic samples. Finally, the gradient penalty term decreased rapidly from 0.8 to ~0.02–0.05 within the first 50 epochs and remained stable thereafter (Figure 7d), confirming satisfaction of the 1-Lipschitz constraint and validating the theoretical assumptions of WGAN-GP.

Together, these metrics indicate that the WGAN-GP training process was stable, well-controlled, and free of mode collapse, providing a reliable foundation for downstream ACmix-Swin classification.

#### 3.6.2. Performance Evaluation of the ACmix-Swin Classifier

**Figure 8:**
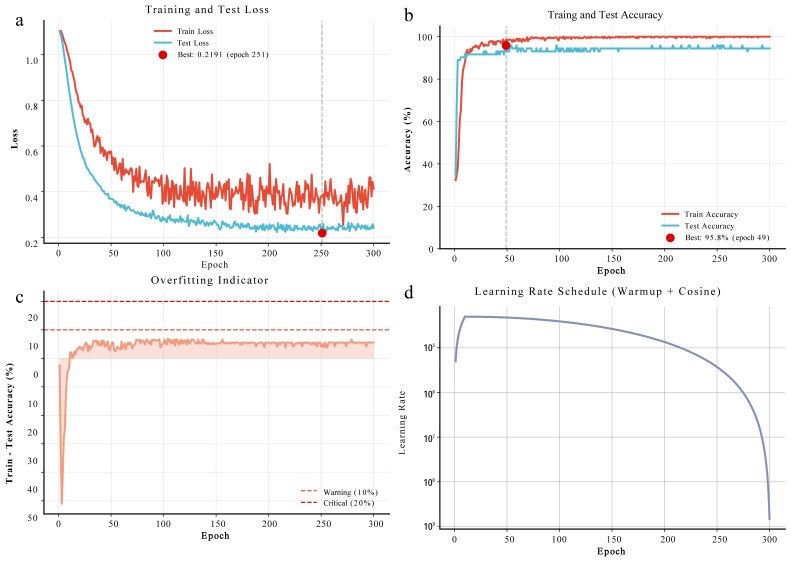
Training dynamics of the ACmix-Swin classifier. (**a**) Training and test loss curves. Both losses decreased rapidly during early epochs, with the lowest test loss (0.2191) reached at epoch 251. Test loss remained consistently below training loss, indicating effective regularization. (**b**) Training and test accuracy. Training accuracy stabilized at 97–99%, while test accuracy peaked at 95.8% and remained stable throughout training. (**c**) Overfitting assessment. The train–test accuracy gap stayed within 3–6%, far below conventional overfitting thresholds, demonstrating strong generalization. (**d**) Learning-rate schedule. A warm-up phase (epochs 0–10) was followed by cosine annealing decay, enabling stable convergence and refined optimization.

The ACmix-Swin classifier demonstrated stable convergence and strong generalization on the augmented transcriptomic dataset (Figure 8). Training and testing losses declined rapidly within the first 50 epochs, with the testing loss reaching its minimum (0.2191) at epoch 251 (Figure 8a). Model accuracy remained consistently high: training accuracy reached 95% within 50 epochs and stabilized at 97–99%, while testing accuracy ranged from 95 to 97% with a peak of 95.8% (Figure 8b). The small train–test accuracy gap (3–6%; Figure 8c) confirms the effectiveness of regularization strategies. The learning rate schedule, combining warmup and cosine decay, further supported smooth optimization (Figure 8d). The final model assigned higher weight to the self-attention branch (0.462) than to the CNN branch (0.365) (Supplemental Figure S1). Overall, ACmix-Swin achieved robust caste classification performance and provides a reliable DL framework for small-sample transcriptomic analysis.

#### 3.6.3. Hub Gene Analysis

**Figure 9:**
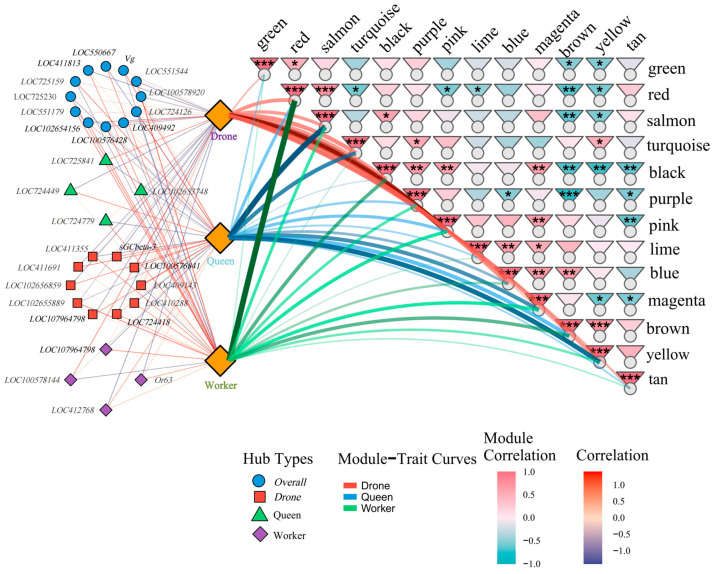
Integrated hub gene selection combining deep learning importance and WGCNA module-trait correlation analysis. (Edges represent gene co-expression strength (thicker lines indicate stronger connectivity), *: *p* < 0.05, **; *p* < 0.01, ***: *p* < 0.001).

To identify key regulators underlying caste differentiation, gene importance scores from the ACmix-Swin model were integrated with WGCNA network topology to construct a combined Hub-gene scoring framework (Combined score = DL_importance × GS × MM) (Supplemental Figure S2). The top 30 candidates per caste were selected based on Gradient × Input importance. Network visualization revealed four Hub-gene groups: overall hubs (blue circles), including the known queen marker *Vg*, and caste-specific hubs for drones (red squares), queens (green triangles), and workers (purple diamonds), each forming distinct subnetwork structures (Figure 9). Module–trait correlations further supported these patterns, with the pink module associated with drones, the salmon and yellow modules with queens, and the red module with workers, indicating cohesive module-level regulatory organization.

Overall, integrating deep learning with WGCNA improved Hub-gene prioritization and uncovered modular regulatory patterns central to caste differentiation. Four selected Hub genes were validated by qPCR, and their expression patterns were consistent with RNA-seq results, supporting the reliability of the screening strategy (Supplemental Figure S3a,b; Supplemental Table S3).

#### 3.6.4. Model Performance Benchmarking

To evaluate model performance, ACmix-Swin was compared with nine commonly used machine learning and deep learning methods, including traditional models (KNN, SVM with RBF kernel, logistic regression, random forest), neural networks (MLP, 1D-CNN, LSTM), and a Transformer architecture. All methods were trained and tested on the same larval expression dataset using five-fold cross-validation.

ACmix-Swin achieved the highest accuracy (95.8%), outperforming all baselines. The best-performing baseline was KNN (82.2%), followed by Transformer (81.1%), and MLP, 1D-CNN, and RBF-SVM (each 80.0%). LSTM (78.9%), logistic regression (77.8%), and random forest (76.7%) showed lower performance. Compared with the strongest baseline (KNN), ACmix-Swin improved accuracy by 13.6 percentage points, corresponding to a 76.4% relative reduction in error rate. It also exceeded Transformer, CNN, and LSTM by 14.7–16.9 points, highlighting the advantage of the hybrid convolution–attention architecture.

**Figure 10:**
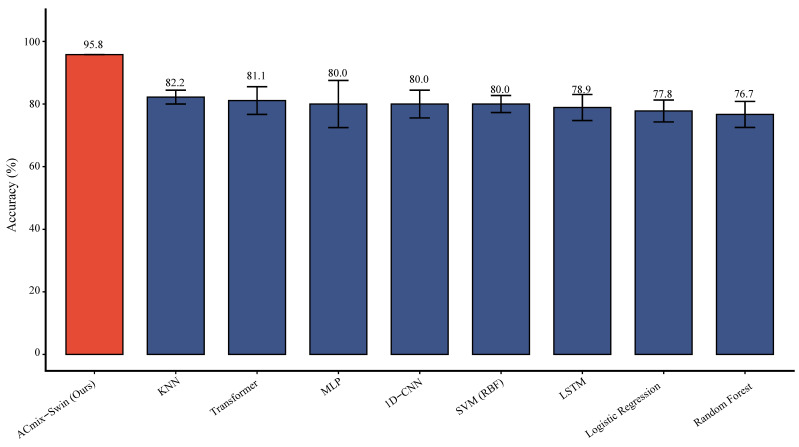
Integrated Analysis of Gene Expression Patterns and Machine Learning-based Caste Classification (The proposed ACmix-Swin model achieves superior classification performance (95.8% accuracy) compared to traditional machine learning approaches.).

Error-bar analysis showed higher variance (2–5%) across folds in baseline models, whereas ACmix-Swin displayed consistently stable performance, indicating superior generalization. Notably, standard deep learning models performed similarly to traditional methods in this task, whereas ACmix-Swin effectively captured both local and global expression patterns (Figure 10; Supplemental Figure S4 and Table S4).

#### 3.6.5. Ablation Analysis of Model Components

**Figure 11:**
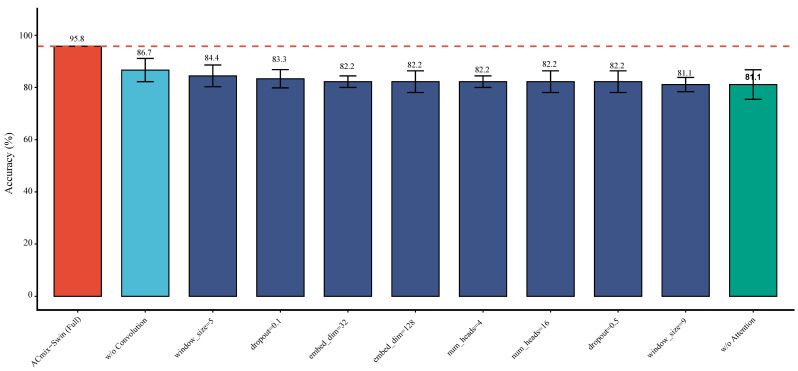
Ablation study demonstrating the contribution of each architectural component in the ACmix-Swin model.

The ablation study systematically assessed the contribution of each component in ACmix-Swin (Figure 11). The full model achieved the highest accuracy (95.8%), outperforming all ablated variants. Removing the convolution branch (86.7%) or the attention branch (81.1%) led to substantial performance drops, confirming the complementary roles of both modules. Modifying window size, embedding dimension, number of attention heads, or regularization strength also reduced accuracy to 80–84%, indicating that the current hyperparameters lie near an optimal configuration. Overall, the ablation results strongly support the necessity and effectiveness of the hybrid convolution–attention architecture (Figure 11; Supplemental Table S5).

## 4. Discussion

To characterize transcriptional differences in 4-day-old *Apis mellifera* ligustica larvae, we reanalyzed RNA-seq datasets from NCBI using a high-quality genome, and mapping statistics confirmed data reliability [47,48,49]. Machine-learning-based dimensionality reduction improved sample separation compared to previous studies (Figure 3). Differential expression revealed early divergence across pathways related to cuticle remodeling, hormone metabolism, sex determination, silk protein synthesis, and reproduction. For example, our differential expression analysis identified a total of 10,740 differentially expressed genes (DEGs), a number substantially higher than that reported in previous transcriptomic studies of honeybee caste differentiation, in which 475 and 687 DEGs were identified in 2-day-old and 4-day-old larvae, respectively [12]. This difference can be partly attributed to the use of an updated reference genome (Amel_HAv3.1 compared with OGSv3.2), as well as more stringent quality control and mapping strategies. More importantly, however, under conventional transcriptomic analysis frameworks, even when large numbers of DEGs are identified, interpretation is typically restricted to a small subset of genes with the largest log_2_foldchanges or the most significant *p*-values. Although well-established caste-associated genes such as Vitellogenin (*Vg*) and Fibroin, which have been repeatedly reported in previous microarray- and RNA-seq–based studies, are consistently recovered, this screening strategy remains insufficient to resolve their relative importance within the global regulatory network. In particular, in the context of high-dimensional transcriptomic data, it remains challenging to determine which genes play dominant regulatory roles, which exhibit functional redundancy, and which minimal gene set constitutes the core discriminative signature distinguishing queen, worker, and drone phenotypes [23]. At the level of developmental timing, our results are highly consistent with those reported previously [12]. Multiple key signaling pathways and regulatory genes previously implicated in caste differentiation, including those involved in hormone biosynthesis, oocyte maturation, and Wnt/Notch signaling, were also identified as significantly differentially expressed in our analysis, further confirming the robustness and reproducibility of earlier findings. Building upon this established developmental framework, the present study extends traditional transcriptomic analyses by introducing a deep learning–based methodology. Unlike approaches that rely solely on statistical significance, the ACmix-Swin model directly couples gene expression patterns with caste classification tasks and prioritizes genes according to their contribution to classification performance. This enables genes to be systematically evaluated based on their discriminative importance for caste identity rather than expression magnitude alone, providing an interpretable ranking of key regulatory candidates and effectively complementing conventional differential expression and functional enrichment analyses [50,51]. Moreover, incorporating WGAN-GP improves robustness under small-sample conditions by learning and simulating high-dimensional expression distributions and expanding the effective training space. This strategy stabilizes feature attribution and facilitates the systematic identification of genes whose absence would substantially impair caste classification, highlighting them as candidate core regulators [52]. Therefore, our model-driven prioritization framework does not negate previous findings; rather, it provides quantitative evidence of central role for established candidates (*Vg*) while simultaneously nominating additional poorly annotated LOC genes as focused targets for downstream functional and network-level validation.

More importantly, many of the high-importance genes prioritized by the ACmix-Swin model map coherently onto the enriched biological pathways, improving accessibility for biologically oriented readers. For example, Vitellogenin (*Vg*), identified as a top-ranked hub gene, is centrally involved in reproductive metabolism, lipid transport, and endocrine regulation, consistent with the strong enrichment of steroid hormone biosynthesis and nutrient metabolism pathways in queens. Likewise, *Or63*, an annotated honeybee odorant receptor, was among the discriminative features and provides a direct molecular anchor to the enrichment of sensory/behavior-related pathways (chemosensory perception and neuroactive ligand–receptor interaction) [53]. Together, these concordant gene–pathway relationships enhance the biological interpretability of model-derived hub genes and support their functional relevance in caste differentiation [54].

Homology annotation indicated that several LOC genes (*LOC102655429*, *LOC726185*, *LOC726094*, *LOC102655476*, *LOC726206*) belong to cuticle protein families. Their strong upregulation in drones suggests early activation of exoskeleton construction and energy allocation programs (Figure 4a,b and Figure 5). GO and KEGG enrichment analyses further supported caste-specific developmental trajectories: drones were enriched for molting, cuticle formation and hormone pathways, while worker–queen differences involved extracellular matrix, neuroactive ligand–receptor interactions, and steroid/retinol metabolism (Figure 6a,b).

We applied the ACmix-Swin deep learning framework to prioritize caste-related regulators among 1874 DEGs. To address the high-dimensional, small-sample characteristics of RNA-seq data, we incorporated WGAN-GP augmentation, which has demonstrated superior stability and fidelity in transcriptomic tasks [30,52,55,56,57,58]. In our dataset, WGAN-GP enabled stable synthetic expression profiles (Figure 8), supporting improved classifier training. Benchmarking confirmed that ACmix-Swin achieved the highest accuracy and F1-score without overfitting (Figure 10 and Figure 11), consistent with best practices for RNA-seq classification [59,60,61].

Four high-importance genes were validated by qPCR, showing expression patterns consistent with RNA-seq (Supplemental Figures S2 and S3a,b), reinforcing the biological reliability of model predictions. This research strategy—combining model-driven feature prioritization with representative experimental validation—has been widely adopted in biomedical studies that integrate WGCNA with machine learning approaches and has been shown to be sufficient for assessing the biological reliability of model-derived feature selection. In such studies, investigators typically validate only a limited number of representative hub genes using qPCR or external datasets to evaluate whether the features identified by the model exhibit stable and reproducible biological signals, rather than performing exhaustive experimental validation of all candidate genes [62,63]. Similarly, methodological studies have emphasized that the primary purpose of qPCR validation is to support the statistical and biological robustness and reproducibility of feature selection strategies, rather than to achieve complete coverage of all candidates [64]. Therefore, this research paradigm itself provides adequate and reasonable experimental support for model-driven gene prioritization results.

Vitellogenin (*Vg*) emerged consistently as the most representative caste-biased gene. Its expression followed a queen > worker > drone pattern (Figure 4c), and queens exhibited the richest alternative splicing events (Supplementary Figure S5a,b; Table S3). Given *Vg*’s pleiotropic roles in reproduction, nutrient allocation, division of labor, lifespan, and immunity [65,66,67,68], it likely serves as a central regulatory hub in early caste differentiation.

Model-prioritized genes (*LOC725841*, *LOC412768*, *LOC100576841*) showed distinct caste-biased expression and may underlie queen energy metabolism, worker task specialization, and drone-specific reproductive or behavioral functions.

In summary, integrating differential expression, co-expression networks, and deep learning allowed us to define core molecular signatures of early caste divergence. The robust candidate genes identified here provide strong targets for future functional studies using RNAi or CRISPR.

## 5. Conclusions

In conclusion, this study integrates genome-guided transcriptome profiling with deep learning to dissect early caste differentiation in 4-day-old *Apis mellifera* larvae. By combining WGAN-GP augmentation, ACmix-Swin classification, differential expression, WGCNA, and splicing analyses, we consistently identified *Vg* and several caste-biased LOC genes as key regulatory candidates. The strong agreement between qPCR and RNA-seq results validates the reliability of our predictions. Overall, our findings show that sex- and caste-related transcriptional programs are established at an early larval stage and highlight the power of deep learning-based feature prioritization for interpreting high-dimensional insect transcriptomes.

## Data Availability

Transcriptome data of 4-day-old *Apis mellifera* larvae were retrieved from the NCBI database, with six biological replicates for each caste (workers: SRS1263211; queens: SRS1263243; drones: SRS1263256).

## Supplementary Materials

The following supporting information can be downloaded at: https://www.mdpi.com/article/10.3390/genes17010017/s1, Supplemental Table S1: Quality of data; Supplemental Table S2: Mapping ratio of data Differentially Expressed Genes Among Drone, Worker, and Queen Larvae Differentially Expressed Genes Among Drone, Worker, and Queen Larvae; Supplemental Table S3: qPCR result; Supplemental Table S4: Compared of Machine Learning; Supplemental Table S5: Ablation comparison; Supplemental Figure S1: ACmix Fusion Weights; Supplemental Figure S2: Top Hub Genes by Phenotype; Supplemental Figure S3: qPCR of hub gene. Supplemental Figure S4: Comparison with Baseline Methods; Supplemental Figure S5a,b: Sasimiplot of *Vg* and qPCR of *Vg* splicing.
