# Supplementary figures and images for: ACmix-Swin Deep Learning of 4-Day-Old Apis mellifera Larval Transcriptomes Reveals Early Caste-Biased Regulatory Hubs
Source: Genes (Basel). 2025 Dec 25;17(1):17. doi: 10.3390/genes17010017 (PMC12841262; doi:10.3390/genes17010017)

b

## Hub gene expression

LOC725841

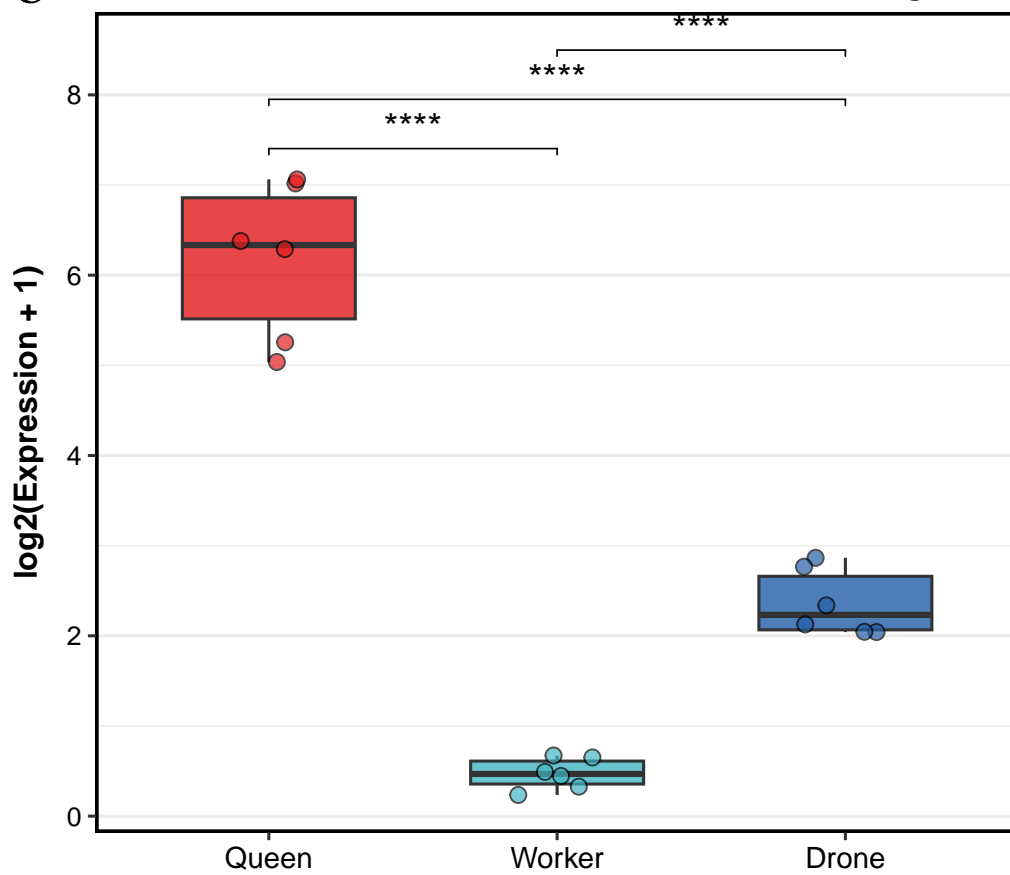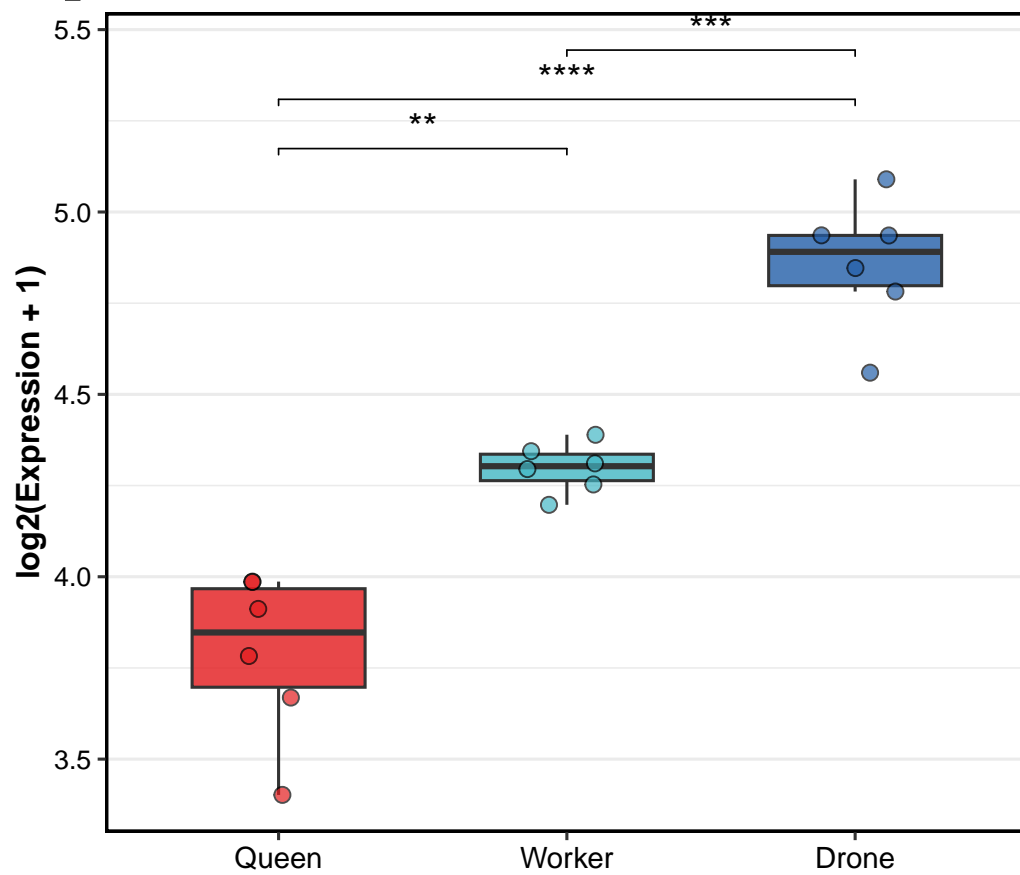

LOC412768

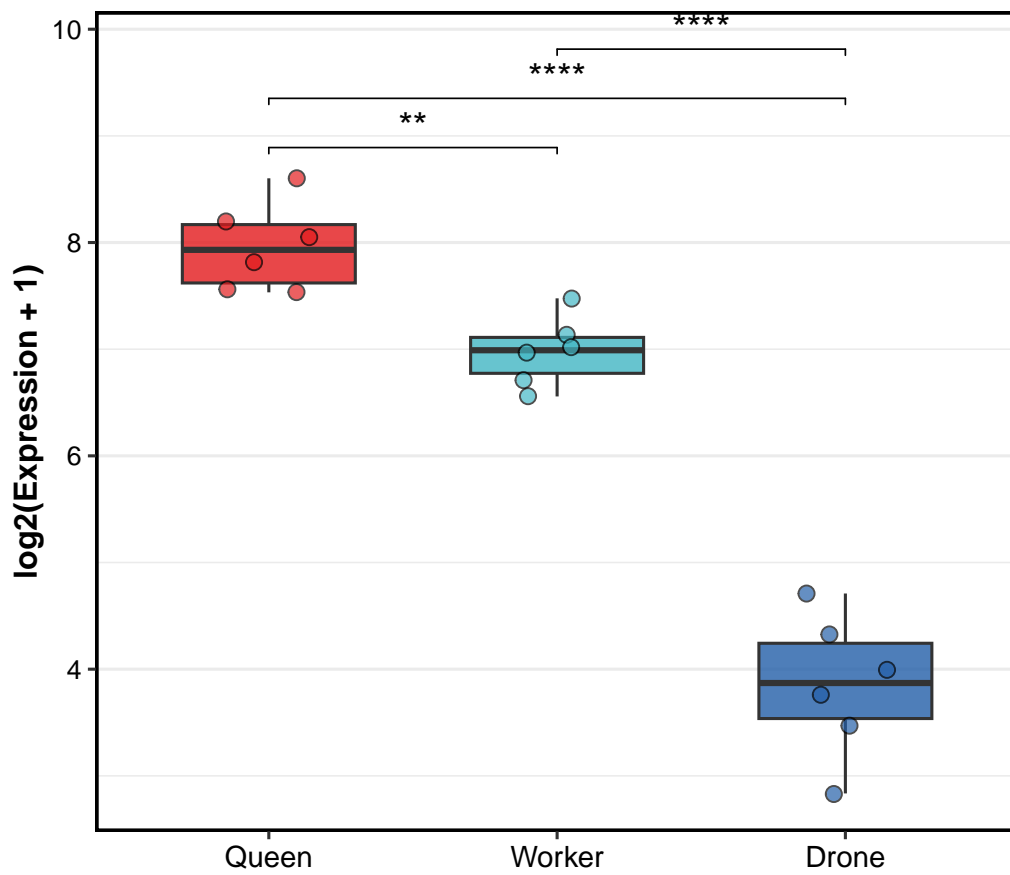

LOC100576841

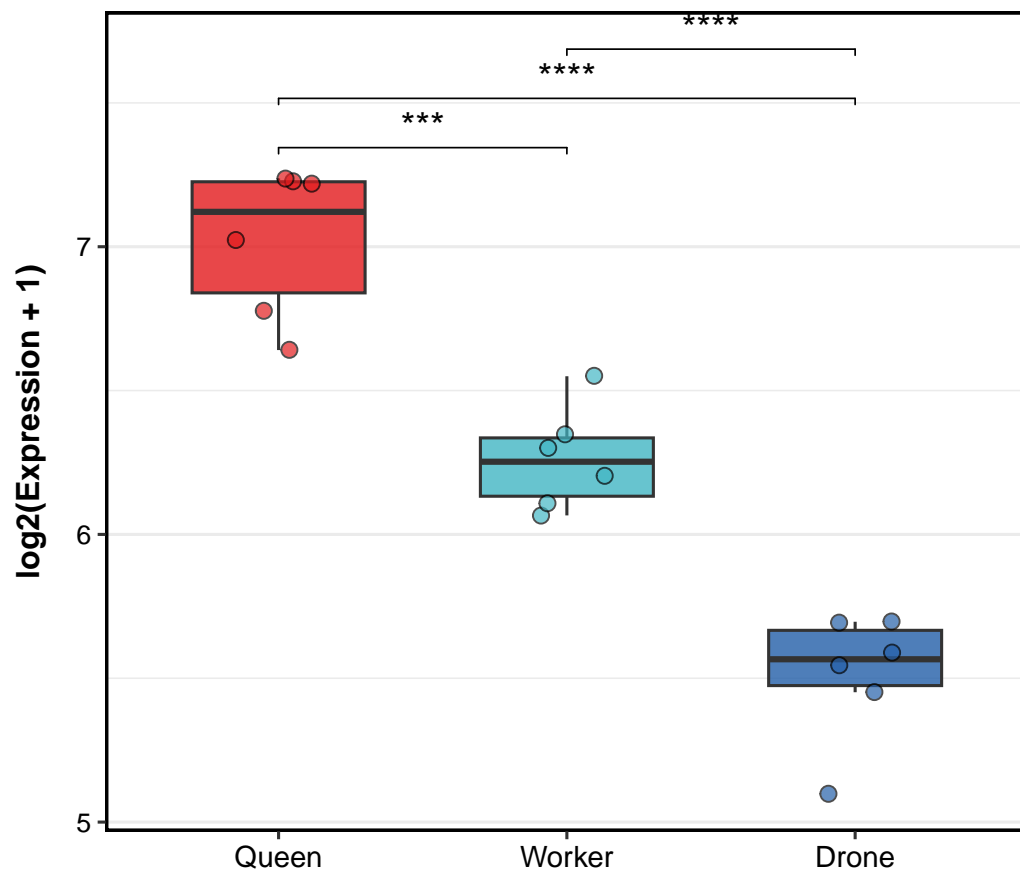

Supplement: Supplementary file 1 [file genes-17-00017-s001.zip › Supplemental/Supplemental Fig3b.pdf]

a

Vg (qPCR)

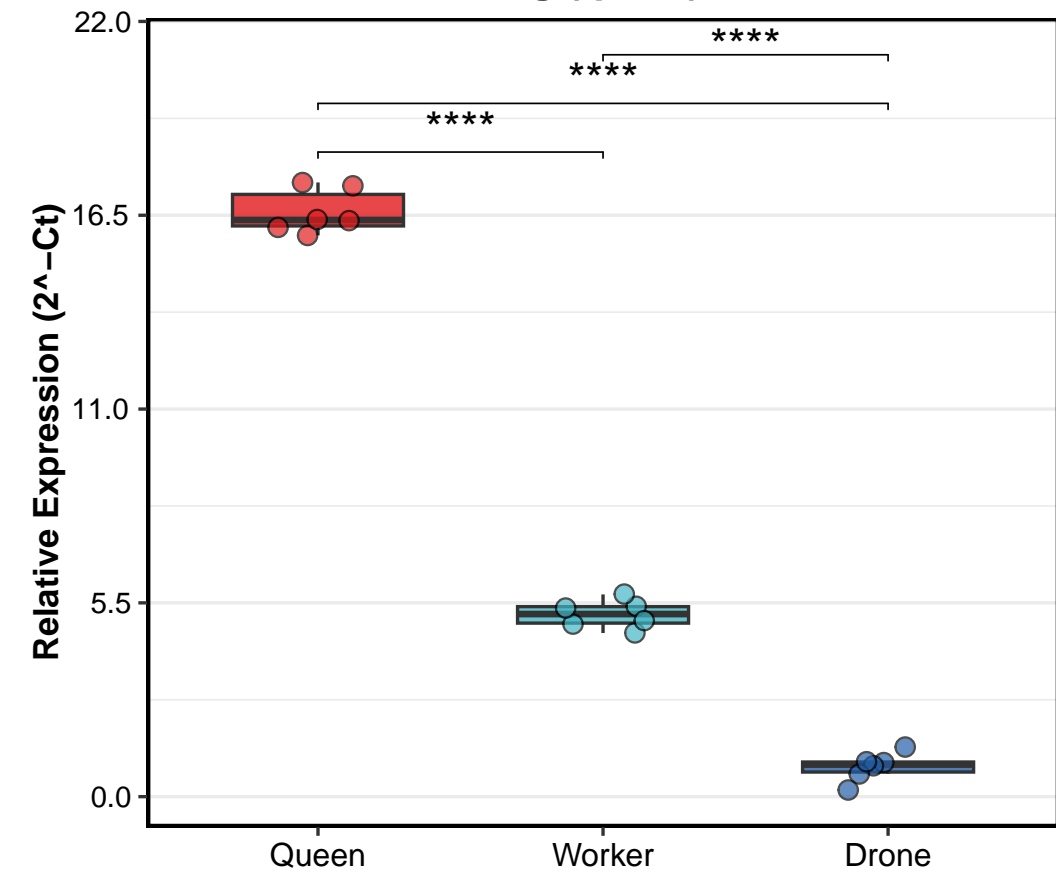

LOC725841 (qPCR)

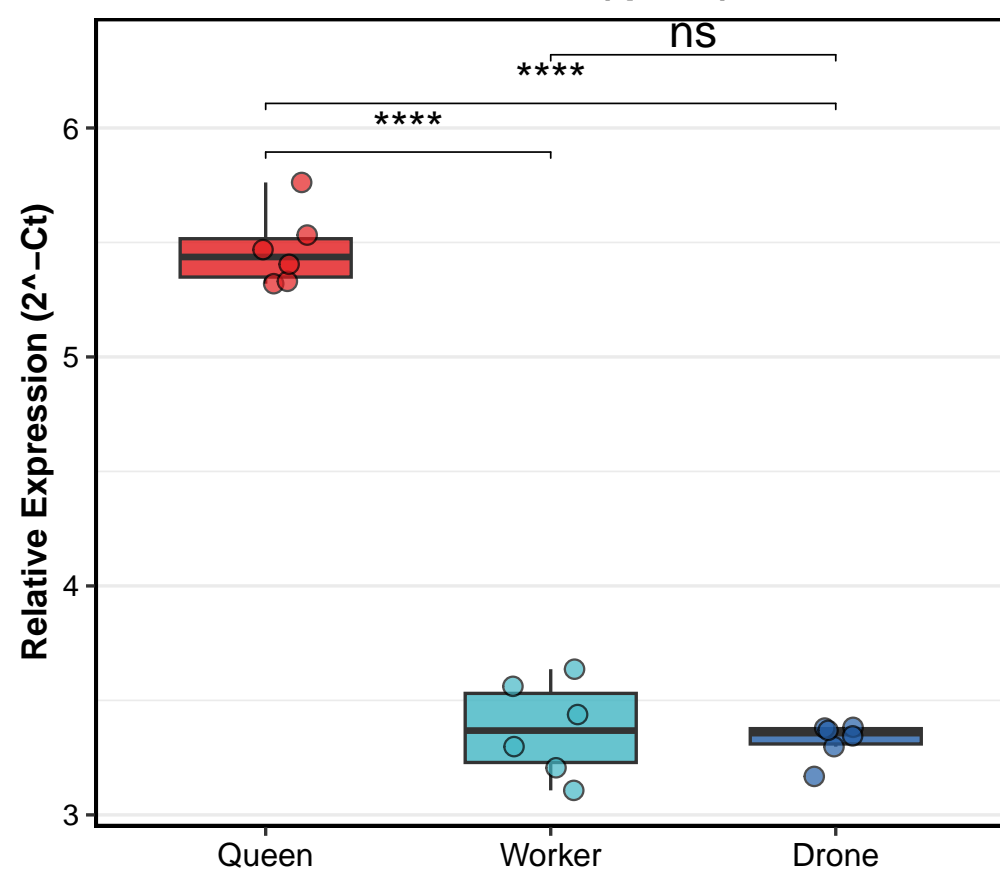

LOC412768 (qPCR)

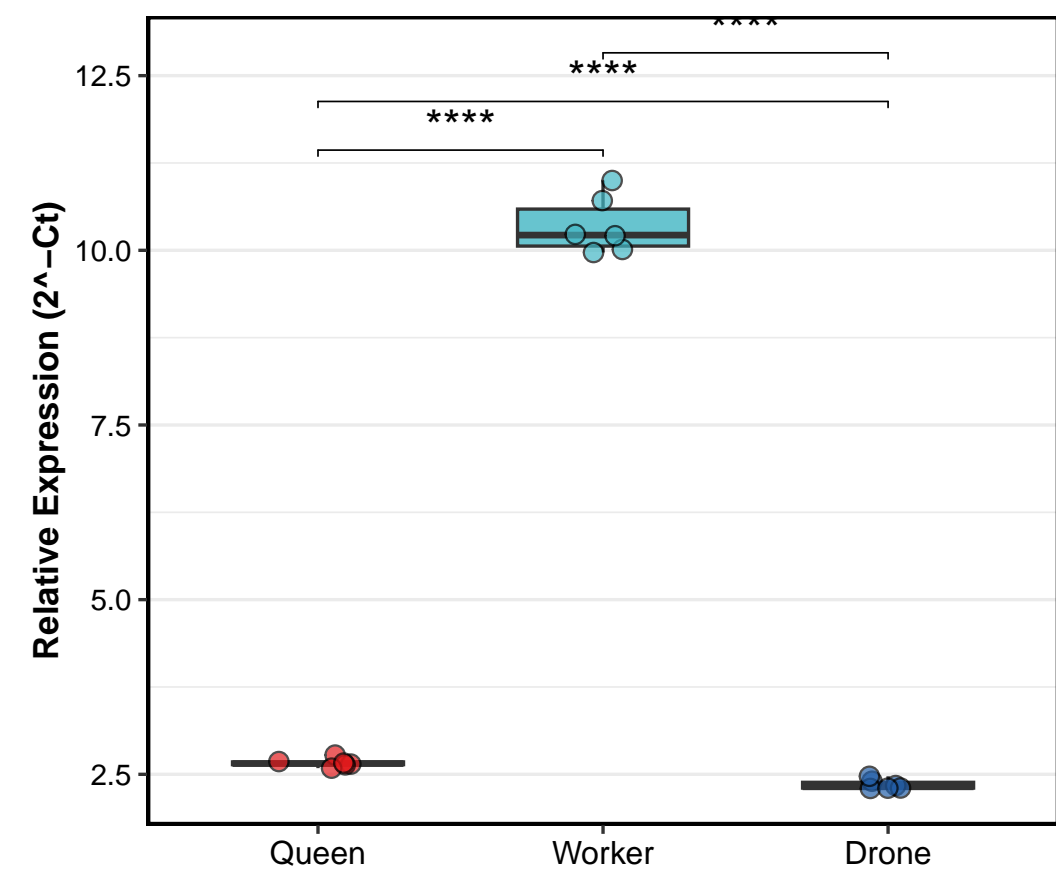

LOC100576841 (qPCR)

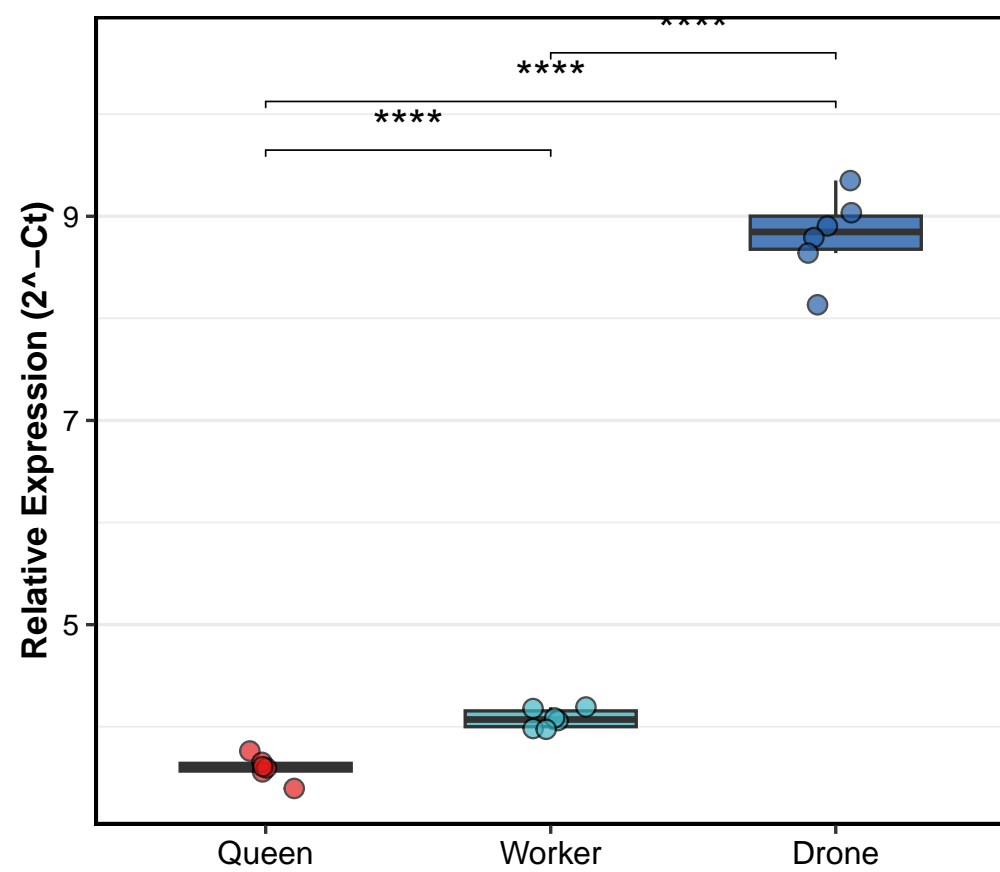

Supplement: Supplementary file 1 [file genes-17-00017-s001.zip › Supplemental/Supplemental Fig3a.pdf]

a

chr4:Vg gene 5028485–5036661

Drone

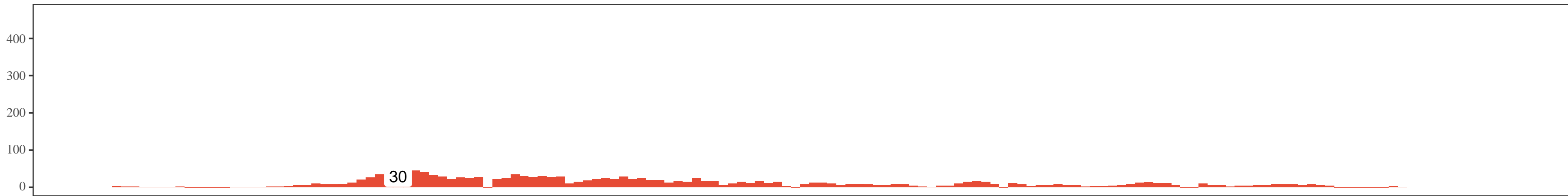

Queen

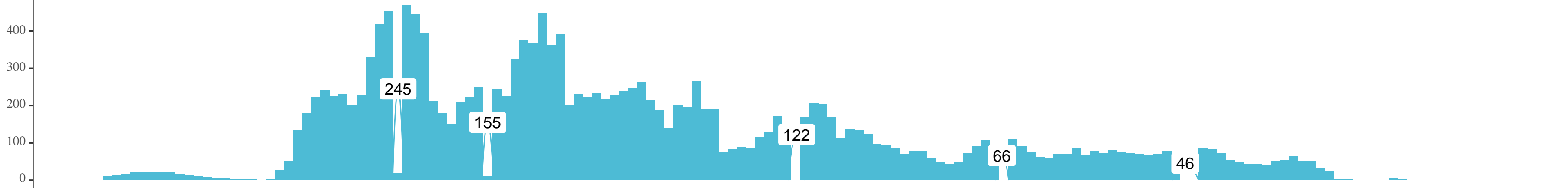

Worker

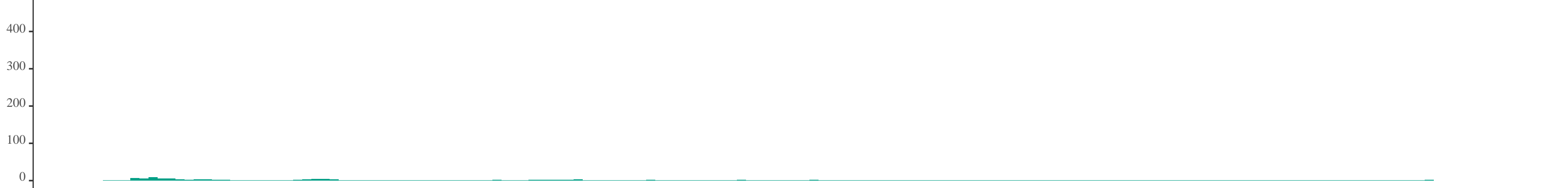

trans

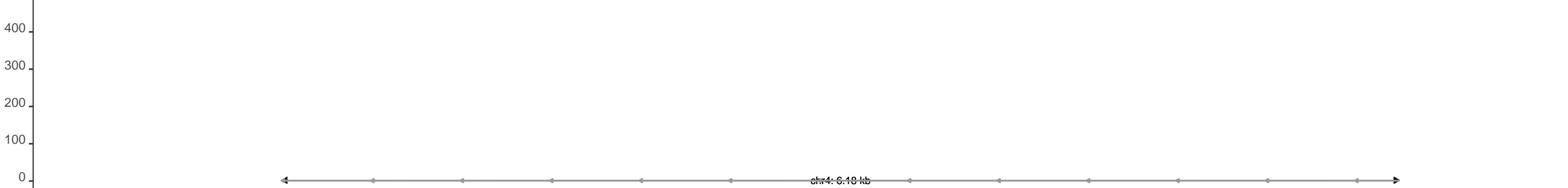

Supplement: Supplementary file 1 [file genes-17-00017-s001.zip › Supplemental/Supplemental Fig5a.pdf]

# Vitellogenin Gene Expression in Honey Bee Castes

b

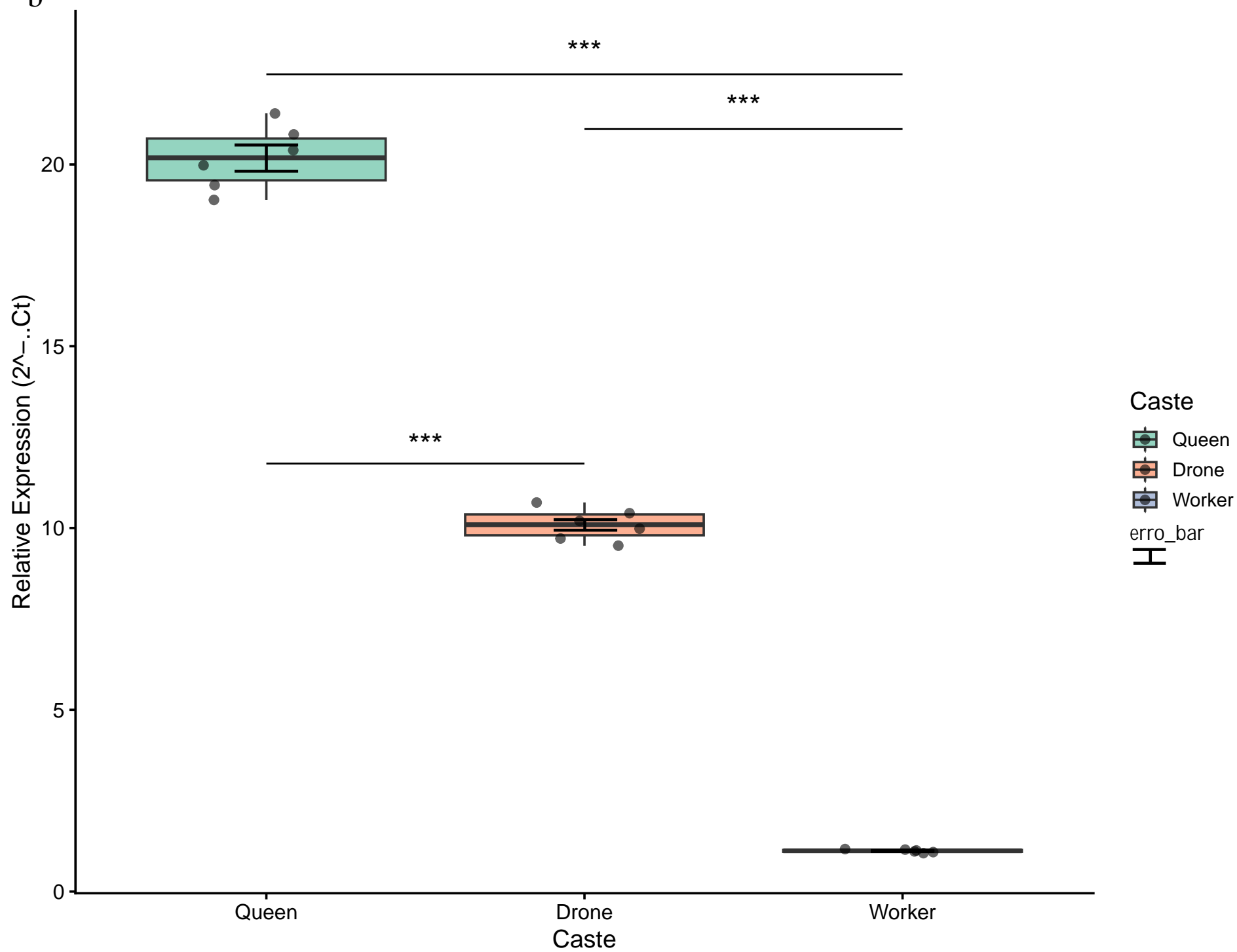

Supplement: Supplementary file 1 [file genes-17-00017-s001.zip › Supplemental/Supplemental Fig5b.pdf]

# Multi-Metric Performance Comparison

**Metric** Accuracy Precision Recall F1-Score

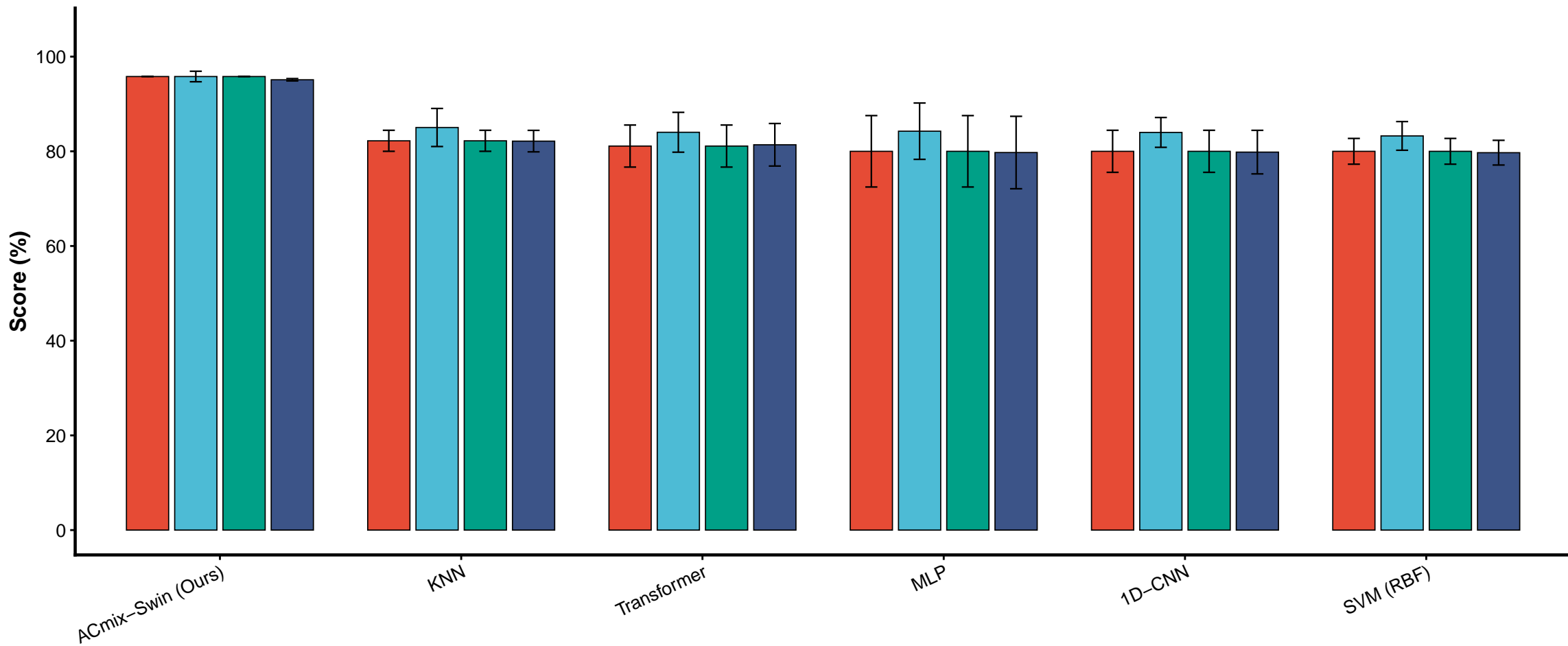

Supplement: Supplementary file 1 [file genes-17-00017-s001.zip › Supplemental/Supplemental Fig4.pdf]

# Top Genes by Phenotype

## Drone

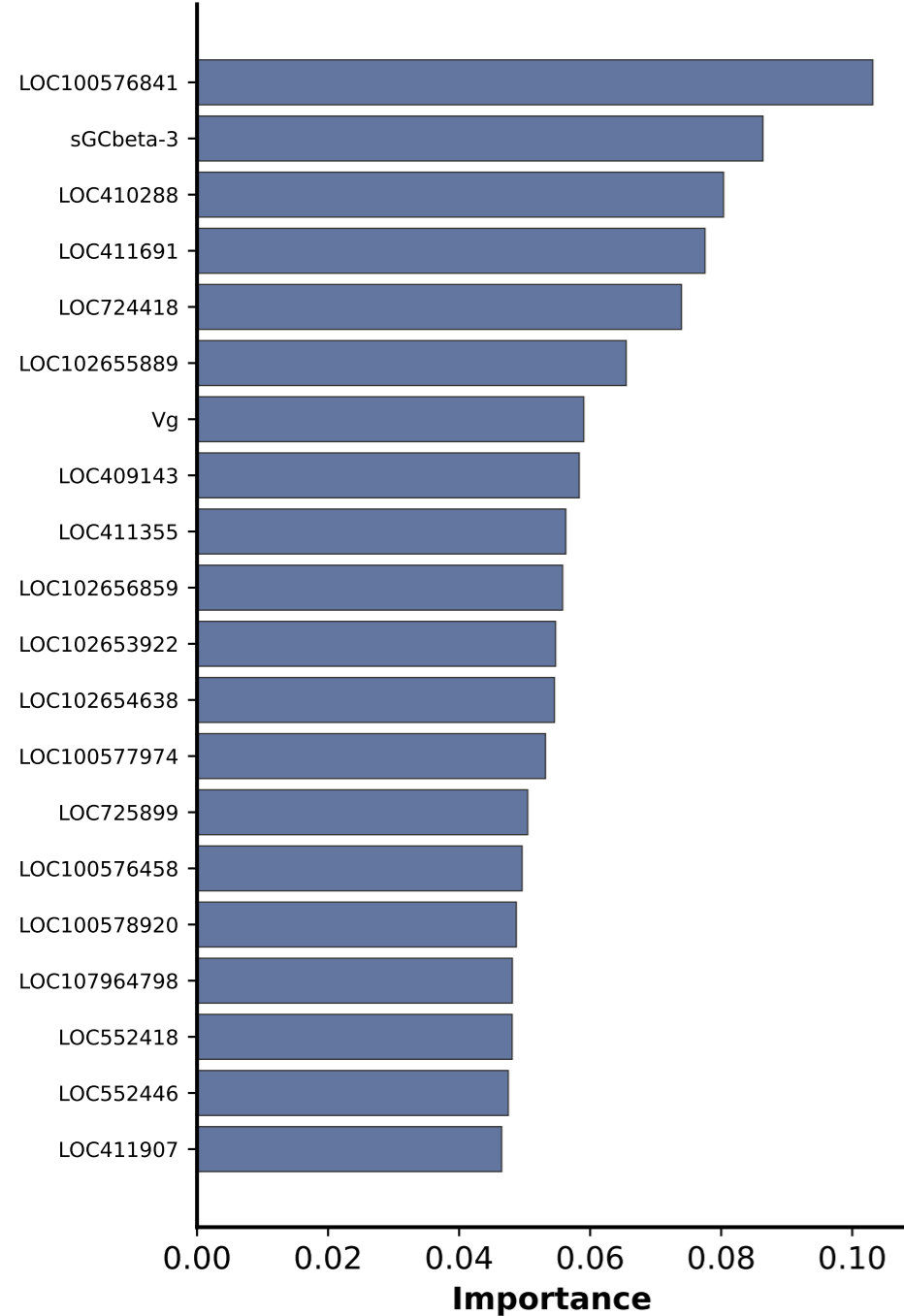

## Queen

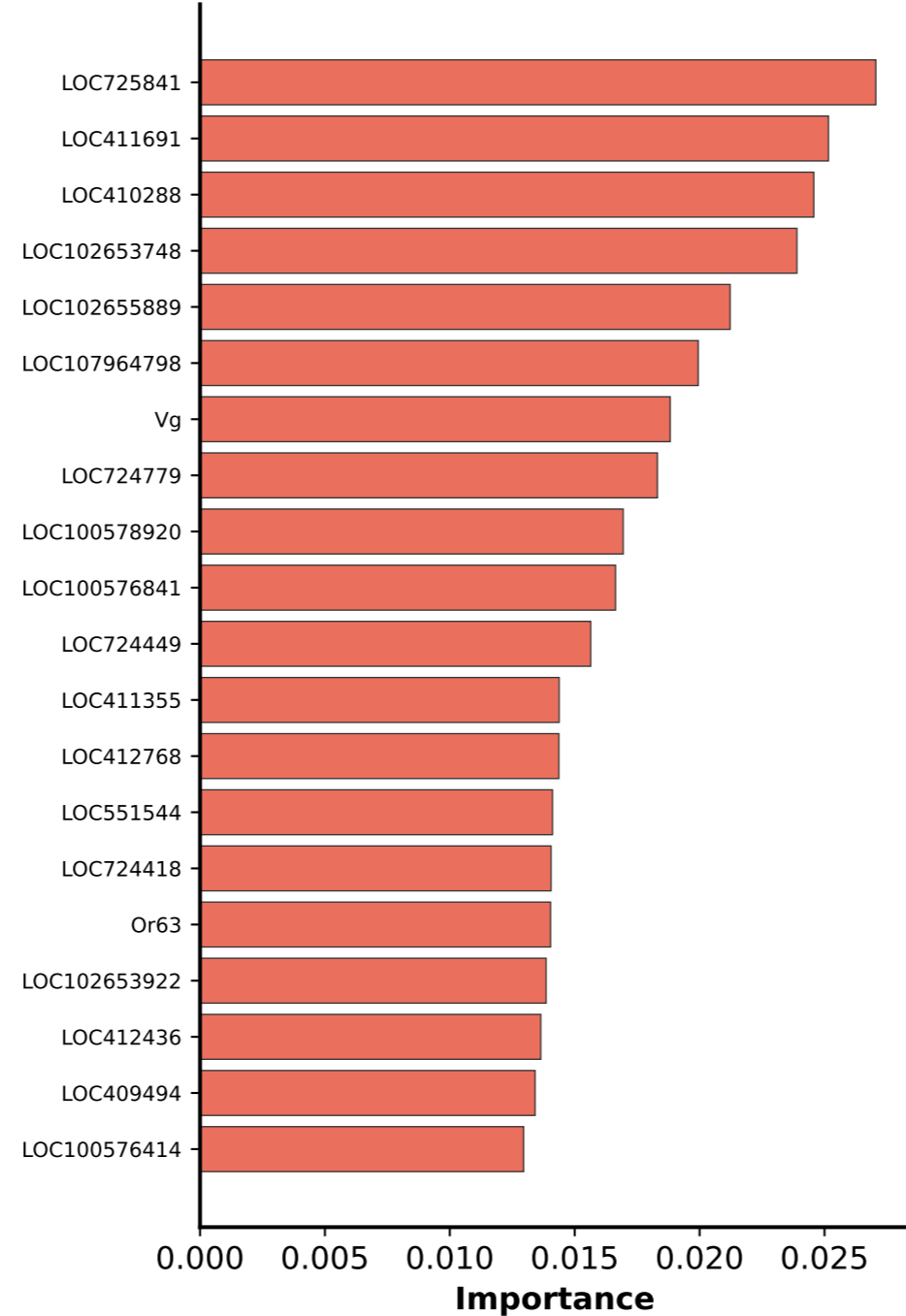

## Worker

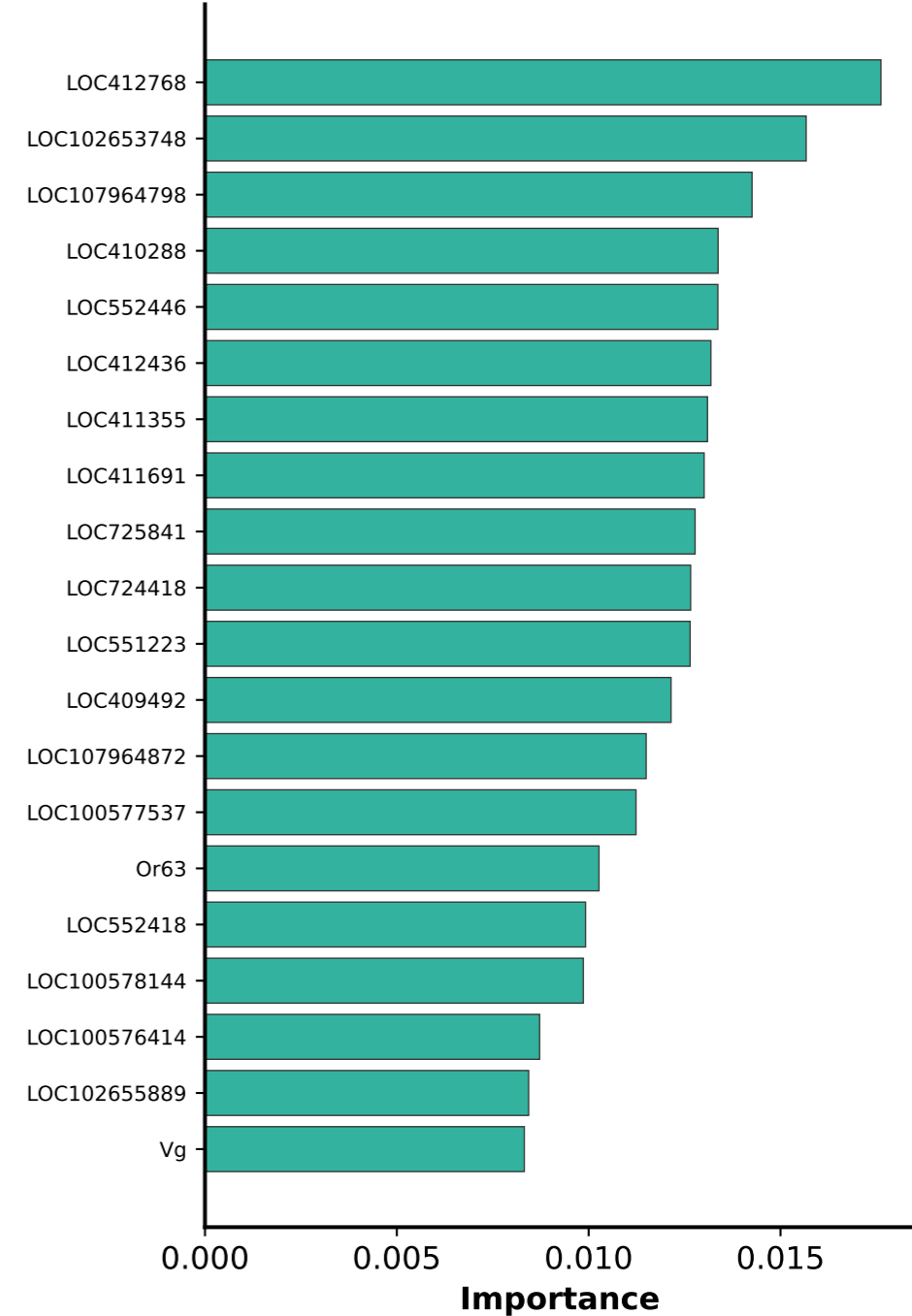

Supplement: Supplementary file 1 [file genes-17-00017-s001.zip › Supplemental/Supplemental Fig2.pdf]

# ACmix Fusion Weights

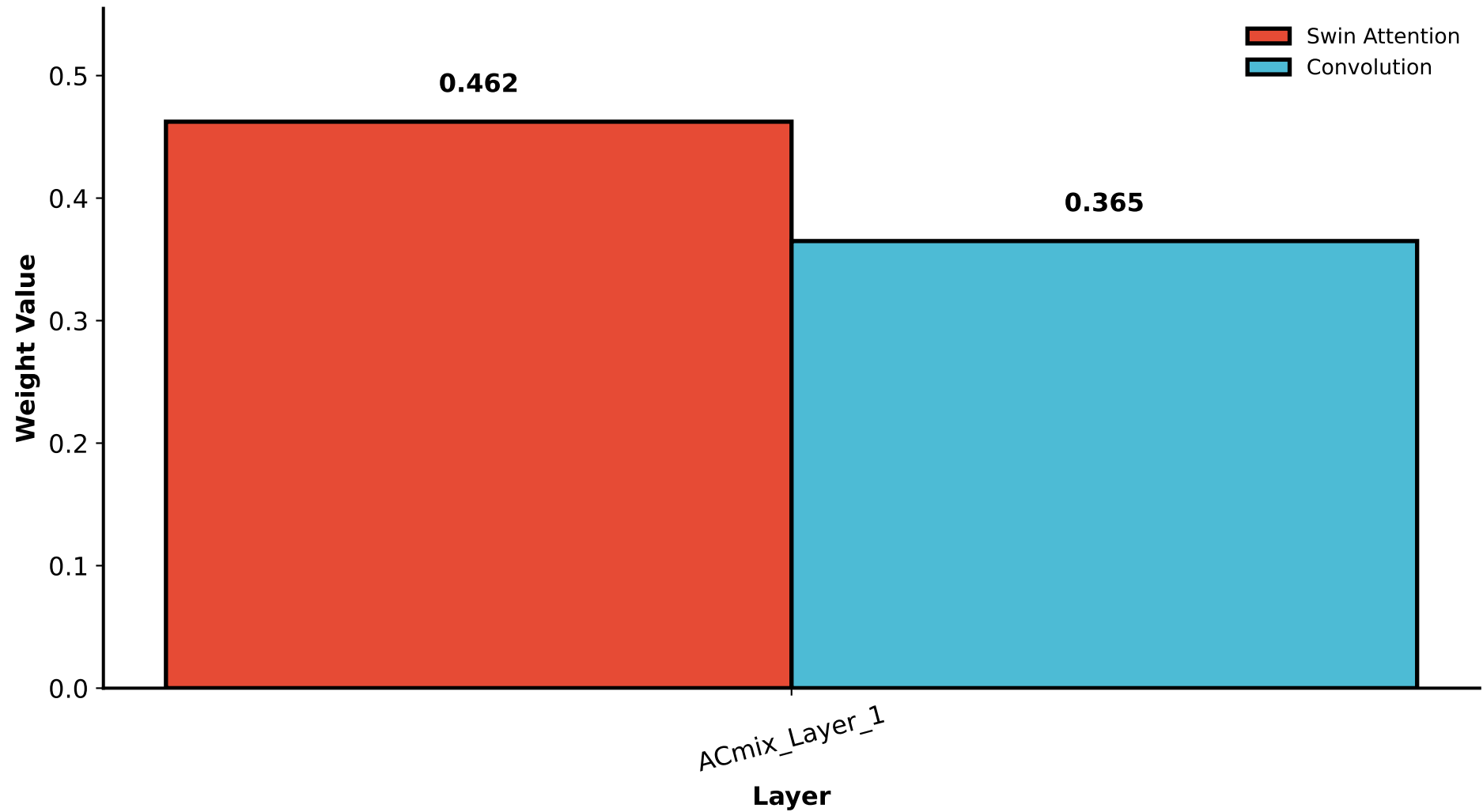

Supplement: Supplementary file 1 [file genes-17-00017-s001.zip › Supplemental/Supplemental Fig1.pdf]
